# Evaluation of MALDI–TOF Mass Spectrometry in Diagnostic and Environmental Surveillance of *Legionella* Species: A Comparison With Culture and *Mip*-Gene Sequencing Technique

**DOI:** 10.3389/fmicb.2020.589369

**Published:** 2020-12-15

**Authors:** Maria Rosaria Pascale, Marta Mazzotta, Silvano Salaris, Luna Girolamini, Antonella Grottola, Maria Luisa Simone, Miriam Cordovana, Francesco Bisognin, Paola Dal Monte, Maria Antonietta Bucci Sabattini, Mariagabriella Viggiani, Sandra Cristino

**Affiliations:** ^1^Department of Biological, Geological, and Environmental Sciences, University of Bologna, Bologna, Italy; ^2^Regional Reference Laboratory for Clinical Diagnosis of Legionellosis, Unit of Microbiology and Virology, Modena University Hospital, Modena, Italy; ^3^Department of Life Sciences, University of Modena and Reggio Emilia, Modena, Italy; ^4^Microbiology Unit-Department of Experimental, Diagnostic and Specialty Medicine, S. Orsola-Malpighi University Hospital, Bologna, Italy; ^5^Regional Agency for Prevention, Environment and Energy of Emilia-Romagna, Bologna, Italy

**Keywords:** *Legionella* non-*pneumophila* species, *Legionella* identification, MALDI-TOF MS, MALDI Biotyper system, agglutination test, *mip*-gene sequencing

## Abstract

*Legionella* spp. are widespread bacteria in aquatic environments with a growing impact on human health. Between the 61 species, *Legionella pneumophila* is the most prevalent in human diseases; on the contrary, *Legionella* non-*pneumophila* species are less detected in clinical diagnosis or during environmental surveillance due to their slow growth in culture and the absence of specific and rapid diagnostic/analytical tools. Reliable and rapid isolate identification is essential to estimate the source of infection, to undertake containment measures, and to determine clinical treatment. Matrix-assisted laser desorption ionization–time-of-flight mass spectrometry (MALDI–TOF MS), since its introduction into the routine diagnostics of laboratories, represents a widely accepted method for the identification of different bacteria species, described in a few studies on the *Legionella* clinical and environmental surveillance. The focus of this study was the improvement of MALDI–TOF MS on *Legionella* non-*pneumophila* species collected during *Legionella* nosocomial and community surveillance. Comparative analysis with cultural and *mip*-gene sequencing results was performed. Moreover, a phylogenetic analysis was carried out to estimate the correlations amongst isolates. MALDI–TOF MS achieved correct species-level identification for 45.0% of the isolates belonging to the *Legionella anisa*, *Legionella rubrilucens*, *Legionella feeleii*, and *Legionella jordanis* species, displaying a high concordance with the *mip-*gene sequencing results. In contrast, less reliable identification was found for the remaining 55.0% of the isolates, corresponding to the samples belonging to species not yet included in the database. The phylogenetic analysis showed relevant differences inside the species, regruped in three main clades; among the *Legionella anisa* clade, a subclade with a divergence of 3.3% from the main clade was observed. Moreover, one isolate, identified as *Legionella quinlivanii*, displayed a divergence of 3.8% from the corresponding reference strain. However, these findings require supplementary investigation. The results encourage the implementation of MALDI–TOF MS in routine diagnostics and environmental *Legionella* surveillance, as it displays a reliable and faster identification at the species level, as well as the potential to identify species that are not yet included in the database. Moreover, phylogenetic analysis is a relevant approach to correlate the isolates and to track their spread, especially in unconventional reservoirs, where *Legionella* prevention is still underestimated.

## Introduction

*Legionella* spp. are Gram-negative bacteria ubiquitous in natural fresh water such as rivers, lakes, and thermal springs. They can also be found in moist soil and mud in which the association with amoebae, protozoa, and biofilms plays a key role in the life cycle of bacteria (Rowbotham, 1980; Fields, 1996; Diederen, 2008).

However, all cases, clusters, or outbreaks are linked to artificial freshwater environments, which represent a main reservoir of *Legionella* and a significant health risk from an epidemiological point of view since the main vehicle of microorganism diffusion and exposure to humans is the aerosol produced by some devices, such as condensers, showers, faucets, humidifiers, whirlpool baths, and medication nebulizer devices (Hines et al., 2014; Mercante and Winchell, 2015; Carlson et al., 2020).

*Legionella* have been found in drinking water distribution systems, cooling towers, and water supply systems, where the water temperature is higher than the environment temperature, sustaining its growth (Diederen, 2008). In fact, *Legionella* grow and replicate between temperatures of 25 and 45°C, with optimal growth between 32 and 42°C (Arvand et al., 2011). In addition, the presence of biofilm in water distribution systems increases the risk of infection, due to the ability of *Legionella* spp. to obtain a high level of nutrients inside biofilm and protection from environmental stresses (Abdel-Nour et al., 2013; Di Pippo et al., 2018). The ability of *Legionella* to replicate inside protozoa cells allows bacteria to then also infect human cells, such as alveolar macrophages, causing the disease in humans known as Legionellosis—an aggressive form of pneumonia—and the Pontiac fever, a febrile and generally benign non-pulmonary disease form (Newton et al., 2010; Mercante and Winchell, 2015; Cunha et al., 2016).

*Legionella pneumophila* (*L. pneumophila*) is the most common and the most studied *Legionella* pathogenic species in humans. This species is divided into 16 serogroups, and the majority of infection cases, confirmed by the use of specific diagnostic criteria (i.e., positive urinary antigen, antibody title movement, and isolation of strain), are attributable to the *L. pneumophila* serogroup 1 (SG1), as confirmed by epidemiological data (Fields et al., 2002; Yu et al., 2002; Beauté, 2017; European Centre for Disease Prevention and Control, 2019; Rota et al., 2019).

However, in addition to *L. pneumophila*, there are more than 60 known species (LPSN Bacterio.net, 2018), some of which are associated with human diseases. Therefore, the risk of infection related to *Legionella* non-*pneumophila* species (Muder and Yu, 2002; Potts et al., 2013; Amemura-maekawa et al., 2018), represents a serious problem in clinical settings (e.g., healthcare facilities, and hospitals), in addition to water for human consumption (Muder and Yu, 2002; World Health Organization, 2007; Decker and Palmore, 2014) and it should not be underestimated.

Among them, *Legionella anisa (L. anisa)* is frequently isolated together with *L. pneumophila* in the hospital plumbing systems and can be used as a probable indicator of epidemic risk, beyond being associated with some cases of human infections (Fallon and Stack, 1990; Fenstersheib et al., 1990; Van Der Mee-Marquet et al., 2006; Vaccaro et al., 2016). Moreover, cases of endocarditis due to *L. anisa* and co-infection in HIV-associated pneumonia have been reported (Compain et al., 2015; Head et al., 2019).

In Australia and New Zealand, *L. longbeachae* isolated from potting soil mixes, represents the main source of human infection with 30.4% of community-acquired Legionellosis (Steele et al., 1990; Yu et al., 2002; Whiley and Bentham, 2011).

Due to its abundance in the environment and the level of pathogenicity, different techniques are applied for the detection and identification of *Legionella* spp., each of which present both advantages and disadvantages.

According to the Italian Guidelines for the prevention and control of Legionellosis (Italian Health Ministry, 2015), the culture technique represents the “gold standard” method for the isolation of *Legionella* strains, as well as for the typing, which is routinely performed by serological and molecular techniques such as the agglutination test, the direct fluorescent antibody (DFA) test, indirect immunofluorescent assay (IFA), sequence-based typing (SBT), and amplification of the macrophage infectivity potentiator (*mip*) gene (Luck, 2002).

In detail during environmental investigations, the isolates from water samples using the culture technique are mainly identified by biochemical and serological tests such as the agglutination test. The agglutination test is routinely used to type *Legionella* isolates, but it is also associated with negative or ambiguous results, leading to inaccurate evaluations of *Legionella* (Orsini et al., 2011). Nevertheless, only an identification at the genus level can be achieved, and in most cases, the quality of the results depends on the experience of the laboratory staff.

Since standard methods are lab-intensive, time-consuming, and prone to delivering false-negative results, several molecular techniques have been developed to detect *Legionella* (Templeton et al., 2003; Blyth et al., 2009).

TheEuropean Working Group for *Legionella* Infection (EWGLI) developed the SBT approach for clinical and environmental *L. pneumophila* strain typing, currently, this tecnique is recognized as the gold standard for the genotyping of *L. pneumophila* strains (Gaia et al., 2005; Ratzow et al., 2007).

Regarding the typing of *Legionella* non-*pneumophila* species, Ratcliff et al. (1998) presented a classification scheme for the *Legionella* genus based on the *mip* gene, encoding for a membrane protein referred to as the “macrophage infectivity potentiator,” that for its genetic stability and specificity is the most reliable and recommended method for identification at the species level (Ratcliff et al., 1997; Fry et al., 2007; Haroon et al., 2012).

Although sensitive and specific, molecular methods are expensive and require specialized laboratories and well-trained staff. Moreover, molecular techniques are affected by their inability to quantify the real risk to humans, as they do not allow for discrimination between dead and alive bacteria, and this leads to a lack of correlation between genomic units and bacterial loads [expressed in colony formant units (CFUs)]. Therefore, the culture-based approach is considered as a reference method in *Legionella* environmental surveillance (Lee et al., 2011; Italian Health Ministry, 2015), although the need to support the culture technique with rapid and cost-effective methods to improve diagnosis and the adoption of the appropriate antibiotic treatment.

In recent years, matrix-assisted laser desorption ionization–time-of-flight mass spectrometry (MALDI–TOF MS) has emerged as an innovative, rapid, and inexpensive technique for species-level microbial identification through the analysis of ribosomal protein patterns. This technique has improved the routine practice of clinical microbiology laboratories, replacing most traditional biochemical or molecular techniques (Maier et al., 2006; Croxatto et al., 2012; Singhal et al., 2015).

Although the use of MALDI–TOF MS is widespread across the world, few data have been published regarding its application for the identification of *Legionella* spp. in clinical and environmental samples (Moliner et al., 2010; Fujinami et al., 2011; Gaia et al., 2011; He et al., 2011; Svarrer and Uldum, 2012; Dilger et al., 2016; Trnková et al., 2018). Moreover, in Italy, to the best of our knowledge, MALDI–TOF MS is not widely used in the clinical or environmental laboratories where *Legionella* surveillance is carried out.

The aim of this study is to evaluate the MALDI Biotyper system for the identification of *Legionella* isolates in the environment. We focused our attention on *Legionella* non-*pneumophila* species isolated from different facilities (i.e., hospitals, healthcare settings, companies, and community areas). Moreover, some of these environments (e.g., homes, companies, and hotels) are supplied by water distribution systems that do not have a water safety plan that includes *Legionella* control measures (i.e., temperature controls, disinfection treatment, and maintenance water system programs), representing a risk of subsequent *Legionella* infections.

The MALDI Biotyper system was compared to other identification methods, such as the agglutination test and *mip*-gene sequencing, to evaluate its performance and its potential use in clinical and environmental *Legionella* surveillance.

## Materials and Methods

The isolates evaluated in this study were collected during the *Legionella* environmental surveillance programs of several municipal water distribution systems, supplying different environments usually associated with risk of *Legionella* infections such as hospitals, healthcare facilities, companies, and community areas (e.g., spas, private apartments, and hotels). The dataset used in the study is shown in Table 1.

**TABLE 1:** Distribution of isolates between man-made environments.

**Number of isolates (*n* = 202)**	**Man-made environments**
121	Hospitals
35	Private apartments
24	Hotels
16	Companies
3	Fitness centers
2	Wellness centers
1	Bathhouses

### *Legionella* Culture and Isolate Selection

Water sampling was performed according to UNI EN International Standard Organization (ISO) 19458:2006 (EN ISO 19458:2006, 2006, 2006 Water quality—Sampling for microbiological analysis) and Italian guidelines (Italian Health Ministry, 2015). Two liters of a hot water sample were collected in sterile polytetrafluoroethylene (PTFE) bottles containing sodium thiosulphate solution (20 mg/L) and then stored at 4°C and processed within 24 h of collection.

The isolation of *Legionella* was performed by the culture technique according to ISO 11731:2017 (ISO 11731:2017, 2017 Water quality—Enumeration of *Legionella*). Briefly, 2 L of the sample was concentrated by filtration on a 0.22-μm polyethersulfone membrane (Sartorius, Bedford, MA, United States). Different aliquots (from 0.2 to 0.1 mL) of the untreated sample or the filtered, heated, and acid-treated sample were seeded on plates of the selective medium glycine–vancomycin–polymyxin B–cycloheximide (GVPC) (Thermo Fisher Diagnostic, Basingstoke, United Kingdom), and incubated at 35 ± 2°C with 2.5% CO_2_ for a maximum of 15 days. During the incubation period, the growth of *Legionella* was evaluated every 2 days, examining the plates for the presence of colonies with specific characteristics ascribable to *Legionella* spp.

Suspected colonies were sub-cultured on buffered charcoal yeast extract (BCYE) agar supplemented with L-cysteine (cys +) and without L-cysteine (cys-) supplementation (Thermo Fisher Diagnostics, Basingstoke, United Kingdom). Positive *Legionella* colonies grow on *Legionella* BCYE cys + agar, but fail to grow on *Legionella* BCYE cys- agar. Furthermore, to provide more evidence that the suspect colonies do not belong to the genus *Legionella*, they were isolated on a blood agar plate (Tryptone Soya Agar + 5% sheep blood) (Thermo Fisher Diagnostics, Basingstoke, United Kingdom): The colonies that grew on blood agar were considered as cysteine non-dependent and were reported as non-*Legionella* spp.

### Serological and Biochemical Typing

A total of 202 isolates of *Legionella* detected in the hot water sample growth on BCYE cys +, without growth on blood agar and with or without a positive reaction to the latex agglutination test, were considered eligible for the study. All colonies grown on BCYE cys +, identified presumably as *Legionella* non-*pneumophila* species, underwent serological typing by the latex agglutination test (*Legionella* latex test kit, Thermo Fisher Diagnostic, Basingstoke, United Kingdom). The test allowed to identify *L. pneumophila* SG1, *L. pneumophila* SG 2–14, and *Legionella* non*-pneumophila* species. Regarding the *Legionella* non*-pneumophila* group, the pool of antibody provided by the manufacturer recognized only a few species most commonly associated with clinical cases, such as *L. anisa*, *L. bozemanii* 1 and 2, *L. gormanii, L. longbeachae* 1 and 2, *L. dumoffii*, and *L. jordanis*.

Three clinical isolates of *L. pneumophila* previously typed as SBT as sequence type 1 (ST1) were included as a positive control. Moreover, five isolates grown on GVPC medium, but which failed to grow on BCYE cys +, were inserted as the negative control. These five isolates were sub-cultured on tryptic soy agar (TSA) (Biolife, Milan, Italy) and the colonies were successively biochemically typed by the Remel RapID NF Plus system (Thermo Fisher Diagnostic) as *Brevundimonas diminuita* (*n* = 1), *Acinetobacter junii* (*n* = 2), and *Flavobacterium lindanitolerans* (*n* = 1), and by Rapid ANA II as *Streptococcus sanguinis* (*n* = 1), according to the manufacturer’s instructions. To confirm the results obtained, the isolates were also analyzed by the MALDI Biotyper system.

### Identification by MALDI–TOF MS

All 202 isolates grown on BCYE cys + with positive, negative, or ambiguous results of the *Legionella* agglutination test were analyzed by the MALDI Biotyper system (Bruker Daltonik GmbH, Bremen, Germany). Spectra acquisition and processing were performed using the Microflex LT mass spectrometer (2,000–20,000 Da, linear positive mode) and the MALDI Biotyper Compass 4.1 software, whose library (version BDAL 7854) included the spectra of 39 *Legionella* strains, as shown in Table 2.

**TABLE 2:** Number of *Legionella* strains spectra included in matrix-assisted laser desorption ionization (MALDI) Biotyper software library.

*L. anisa* (*n* = 9)	*L. feeleii* (*n* = 8)	*L. longbeachae* (*n* = 9)	*L. rubrilucens* (*n* = 1)
*L. beliardensis* (*n* = 1)	*L. geestiana* (*n* = 1)	*L. maceachernii* (*n* = 3)	*L. sainthelensi* (*n* = 3)
*L. birminghamensis* (*n* = 6)	*L. gormanii* (*n* = 2)	*L. micdadei* (*n* = 5)	*L. santicrucis* (*n* = 3)
*L. bozemanii* (*n* = 10)	*L. gratiana* (*n* = 5)	*L. moravica* (*n* = 1)	*Legionella* sp. (*n* = 1)
*L. brunensis* (*n* = 2)	*L. hackeliae* (*n* = 1)	*L. oakridgensis* (*n* = 2)	*L. tucsonensis* (*n* = 2)
*L. cherrii* (*n* = 4)	*L. impletisoli* (*n* = 1)	*L. parisiensis* (*n* = 1)	*L. wadsworthii* (*n* = 1)
*L. cincinnatiensis* (*n* = 2)	*L. israelensis* (*n* = 1)	*L. pneumophila* (n = 3)	*L. waltersii* (*n* = 1)
*L. dresdenensis* (*n* = 1)	*L. jamestowniensis* (*n* = 1)	*L. pneumophila* ssp *fraseri* (*n* = 4)	*L. worsleiensis* (*n* = 1)
*L. dumoffii* (*n* = 7)	*L. jordanis* (*n* = 2)	*L. pneumophila* ssp *pascullei* (*n* = 2)	*L. yabuuchiae* (*n* = 1)
*L. erythra* (*n* = 1)	*L. lansingensis* (*n* = 1)	*L. pneumophila* ssp *pneumophila* (*n* = 6)	

During the first part of the experiments a comparison between direct smear and full extraction methods was performed for a subset of strains (20/202, 10%), following manufacturer’s instructions. No significant differences were found in term of identification and quality of spectra. Therefore, considering that *Legionella* is a Gram-negative bacterium, and the full extraction method is recommended for species with thick cell wall, such as Gram-positives, *Actinomycetales*, and fungi, the direct smear method was applied on the remain *Legionella* isolates.

Briefly, a small amount of bacterial biomass, picked from a fresh plate culture (24–48 h of incubation) was spotted in duplicate onto a MALDI Biotyper target, overlaid with 1 μl of MALDI Biotyper matrix solution, and allowed to air dry before the measurement. The identification result was considered reliable when the log (score) was ≥ 2.0 (“high confidence level”) or between 1.7 and 1.99 (“low confidence level”). In addition, in case of log (score) results between 1.4 and 1.59, identification was considered reliable when the first four proposed results were identical (Christner et al., 2010; Schubert et al., 2011). Identification was considered reliable also in those cases in which the difference in the log (score) values between the first- and second-best matches was ≥ 0.3 (Martiny et al., 2012). In contrast, the presence of score of 0.00–1.69 indicated a non-reliable identification.

Determination of the sensitivity, specificity, and confidence interval (CI) at the 95% level of significance was performed using GraphPad Prism software version 8.0.1 for Windows (GraphPad Software, San Diego, California, United States).

### Identification of *Legionella* spp. by *mip*-Gene Sequencing

The DNA extraction was carried out using the InstaGene Purification Matrix (Bio-Rad, Hercules, CA), and DNA concentrations were determined using the Qubit fluorometer (Thermo Fisher Scientific, Paisley, United Kingdom). The PCR for all *Legionella* non-*pneumophila* species isolates was performed using the protocols for the *mip* gene suggested by EWGLI, as described by Ratcliff et al. (1998). The *mip-*gene amplification was carried out using degenerate primers and modified by M13 tailing to avoid noise in the DNA sequence (Mentasti et al., 2012). The *mip-*gene amplification was carried out in a 50-μL reaction containing DreamTaq Green PCR Master Mix 2 × (Thermo Fisher Diagnostic Basingstoke, United Kingdom) and 40 picomoles of each primer; 100 ng of the DNA extracted from the presumptive colonies was added as template.

Specifically, the *mip* amplicons were sequenced using tailed M13 forward and reverse primers (*mip*-595R-M13R caggaaacagctatgaccCATATGCAAGACCTGAGGGAAC and *mip*-74F-M13F tgtaaaacgacggccagtGCTGCAACCGATGCCAC) to obtain a complete coverage of the sequenced region of interest (Mentasti et al., 2012). Amplification was performed in a thermocycler under the following conditions: Pre-denaturation for 3 min at 96°C, then 35 cycles consisting of 1 min at 94°C for denaturation, 2 min at 58°C for annealing, and 2 min at 72°C for extension, followed by a final extension at 72°C for 5 min. The reaction mixtures were then held at 4°C.

The PCR products were visualized by electrophoresis on 2% agarose gel and stained with ethidium bromide. Following purification, DNA was sequenced using BigDye Chemistry and analyzed on an ABI PRISM 3100 Genetic Analyzer (Applied Biosystems, Foster City, CA).

Raw sequencing data were assembled using CLC Main Workbench 7.6.4 software. The sequences were compared to sequences deposited in the *Legionella mip*-gene sequence database using a similarity analysis tool. EWGLI have established an accessible web database^1^ that contains sequence data from described species and allows the identification of *Legionella* non-*pneumophila* species. The species-level identification as done on the basis of ≥ 98% similarity to a sequence in the database (Fry et al., 2007). For one strain with a sequence generically identified in the *mip*-gene sequence database as *Legionella* species (*L*. spp.), the sequence was also queried in the GenBank database using the basic local alignment search tool (BLAST), and it showed the best match with *Legionella species* H (*L. species* H), (Ratcliff et al., 1998). The 205 nucleotide *mip* sequences generated for this study were submitted to GenBank. The provided accession numbers were as follows: MW021138, MW052863-MW053066.

### Phylogenetic and Allelic Diversity Analysis

To estimate the relationship among the *Legionella* species found, a multiple sequence alignment (MSA) and a phylogenetic tree were performed on the 202 *mip*-gene sequences and the three positive controls. For each taxon, identified as previously described, the reference *mip* sequence of the correspondent American Type Culture Collection (ATCC) and Institute of Medical and Veterinary Science (IMVS) strain was retrieved and added to the analysis. When required, a manual editing was performed on the sequences, trimming them to the same length as the reference sequence. The nucleotide sequences were aligned by the multiple sequence comparison by log-expectation (MUSCLE) program (Edgar, 2004), executed in Geneious Prime 2020.1.2^2^, retaining the default settings. The phylogenetic tree was built by the Geneious Tree Builder, using Tamura–Nei (Tamura and Nei, 1993) as a genetic distant model and neighbor-joining (Saitou and Nei, 1987) as a tree building method, and then bootstrapped using 100 replicates.

## Results

### *Legionella* Identification by the Agglutination Test

**FIGURE 1:**
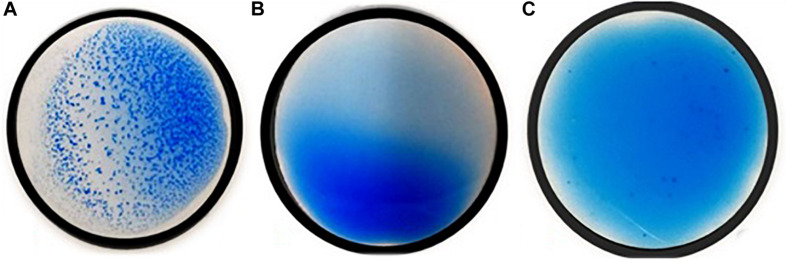
Positive **(A)**, negative **(B)**, and ambiguous **(C)** *Legionella* non*-pneumophila* species results of the agglutination test.

Overall, 34/202 (16.8%) isolates resulted positive and 158/202 (78.2%) negative in the agglutination test, while 10/202 (5.0%) isolates provided an ambiguous result. All of the strains that were included as controls delivered the expected results. Examples of the agglutination results are shown in Figure 1.

### MALDI Biotyper System Results

Applying the cut-offs recommended by the manufacturer, the MALDI Biotyper system identified at the genus level 90/202 (44.5%) isolates. Among them, 59/90 (65.5%) were identified at the species level with a high confidence level (*n* = 50 *L. anisa; n* = 1 *L. feeleii;* and *n* = 8 *L. rubrilucens*), and 31/90 (34.4%) were identified at the genus level with a low confidence level (*L. anisa*, *L. feeleii*, *L. jordanis*, and *L. rubrilucens*; Table 3); 112/202 isolates (56.0%) remain without identification. The application of the additional interpretation criteria for identification results with a log (score) ≤ 1.69 enabled a further 40 strains to be identified from 112 un-identified isolates, achieving a total of 130/202 (64.4%) successful results. In contrast, 72 isolates remain unidentified. The summary of the MALDI Biotyper system results is shown in Table 3.

**TABLE 3:** Isolates identification by MALDI Biotyper system according to the manufacturer’s threshold and the revised sub-criteria.

**Identification**	**Isolates**	**Manufacturer thresholds and number *(n)* of isolates**			**Total manufacturer identification (*n*)**	* Sub-Criteriafor score ≤ 1.69	**Total(*n*)**
					
		2.00–3.00	1.70–1.99	0.00–1.69 *			
*Legionella*	*L. anisa*	50	2		52		52
	*L. feeleii*	1	8		9	2*	11
	*L. jordanis*		1		1		1
	*L. rubrilucens*	8	20		28	38*	66

	**Total**	59	31		90	40*	130

Not Identified*				112 *	112	72	72
Total					202		202

*(Christner et al., 2010; Schubert et al., 2011; Martiny et al., 2012).

The isolates used as controls were identified with a high confidence level as *L. pneumophila* and other bacteria (i.e., *Acinetobacter junii*, *Brevundimonas dimunita*, *Flavobacterium lindanitolerans*, and *Streptococcus sanguinis*).

### Comparison Between the Agglutination Test and MALDI Biotyper System Results

The 34 isolates with positive agglutination results were identified by the MALDI Biotyper system as *L. anisa*. The main isolates involved in the study (*n* = 158) displayed negative agglutination results, of which the MALDI Biotyper identified 89/158 (56.3%) of the isolates as *Legionella genus*, while the other 69/158 (43.7%) were not identified.

Regarding the isolates with ambiguous agglutination results (*n* = 10), the MALDI Biotyper system correctly identified *Legionella* in seven cases (*n* = 4 *L anisa* with a high score, and *n* = 3 *L. rubrilucens* with a revised low score); in contrast, 3/10 isolates were not identified.

Full concordance between the two techniques was found for the control strains. However the three positive controls identified by the agglutination reaction as *L. pneumophila* SG1, 3, and 6 were not distinguished at the serogroup level by the MALDI Biotyper system.

### *mip*-Gene Sequencing

The results obtained by *mip*-gene sequencing with the respective range of matches to the reference strains (i.e., ATCC, IMVS-911, and IMVS-3376) are shown in Table 4.

**TABLE 4:** Results of the *mip-*gene sequencing and the number of isolates identified.

***mip-gene* sequencing results**	**Isolates identified ***n* (%)****	**% of match with reference strains**
*L. anisa*	50 (24.8%)	100%
	2 (1.0%)	96.7%
*L. feeleii*	1 (0.5%)	99.4%
	1 (0.5%)	98.4%
	9 (4.5%)	98.2%
*L. jordanis*	1 (0.5%)	100%
*L. londiniensis*	7 (3.5%)	100%
*L. nautarum*	15 (7.4%)	100%
*L. quateirensis*	4 (1.9%)	98.2%
*L. quinvilanii*	1 (0.5%)	96.2%
*L. rubrilucens*	30 (14.9%)	100%
*L. species* H	1 (0.5%)	100%
*L. steelei*	1 (0.5%)	99.8%
*L. taurinensis*	79 (39.1%)	100%
**TOTAL**	**202 (100%)**	

The most common species detected by the sequencing analysis were *L. taurinensis* (39.1%), followed by *L. anisa* and *L. rubrilucens* (Table 4). Among the *L. anisa* subset, two isolates, identical to each other, were found to match the reference sequence strain at 96.7%, showing 20 mismatches with the main *L. anisa* group.

Only one isolates generically identified as *L.* spp. In the *mip*-gene database, were matched in GenBank and identified as *L. species* H (Ratcliff et al., 1998). The positive control was confirmed to belong to *L. pneumophila*; in contrast, the negative controls were not included in the *mip*-gene sequences analysis.

### Comparison Between the MALDI Biotyper System and *mip*-Gene Sequencing Results

Among the species included in the MALDI Biotyper system database, the concordance between this system and the sequencing was 91/94 (96.9%), as follows: *L. anisa* (*n* = 52/52), *L. rubrilucens* (*n* = 27/30), *L. feeleii* (*n* = 11/11), and *L. jordanis* (*n* = 1/1). Therefore, among the species included in the MALDI Biotyper system database, the overall sensitivity of this system was 96.8% (95% CI: 0.91–0.99).

In the remaining 108 isolates identified by *mip*-gene sequencing as *L. londiniensis*, *L. nautarum*, *L. quateirensis*, *L. quinlivanii, L. species* H, *L. steelei*, and *L. taurinensis*, whose spectra are not included in the MALDI Biotyper system database, the MALDI Biotyper system did not identify 69 isolates and led to a misidentification in 39 cases (*L. rubrilucens* instead of *L. taurinensis*). Therefore, the specificity of the MALDI Biotyper system was 69/108 = 63.9% (95% CI: 0.55–0.72).

A comparison between results delivered by the three techniques evaluated in this study is shown in Table 5.

**TABLE 5:** Comparison of the results of the three techniques.

***Mip*-gene sequencingresults**	**Latex agglutination test results**			**MALDI Biotyper system results**		
		
Isolate identification (*n*)	positive (+), negative (–), and ambiguous (±) results (*n*)					
	** +**	–	** ±**	**2.00–3.00(*n*)**	**1.70–1.99(*n*)**	0.00–1.69 *(*n*)
*L. anisa* (52)	**34**	14	4	***L. anisa*(50)**	***L. anisa*(2)**	
*L. feeleii* (11)		11		***L. feeleii*(1)**	***L. feeleii*(8)**	***L. feeleii*(2)**
*L. jordanis* (1)		1			***L. jordanis*(1)**	
*L. londiniensis* (7)		5	2			Not Identified (7)
*L. nautarum* (15)		14	1			Not Identified (15)
*L. quateirensis* (4)		4				Not Identified (4)
*L. quinvilanii* (1)		1				Not Identified (1)
*L. rubrilucens* (30)		30		***L. rubrilucens*(8)**	***L. rubrilucens*(17)**	***L. rubrilucens*(2)** Not Identified (3)
*L. species* H (1)		1				Not Identified (1)
*L. steelei* (1)		1				Not Identified (1)
*L. taurinensis* (79)		76	3		*L. rubrilucens* (3)	*L. rubrilucens* (36) Not Identified (40)

* Manufacturer’s criteria plus sub-criteria (Christner et al., 2010; Schubert et al., 2011; Martiny et al., 2012). In bold type the concordance between techniques.

### Phylogenetic Analysis

**FIGURE 2:**
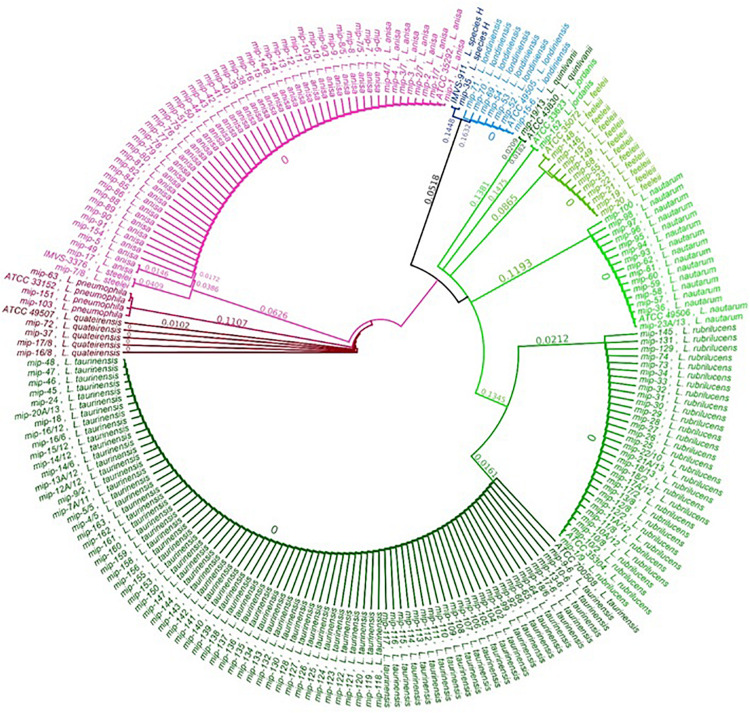
Phylogenetic tree obtained by the *mip* sequence analyses of isolates and strain types.

The relationship between the 202 *Legionella* isolates, positive controls, and reference strains was studied by a phylogenetic tree of the sequenced *mip* gene in order to display a graphical representation of the inter- and intra-specific genetic variability among the taxa previously identified (Figure 2). The phylogenetic tree shows that the clusters corresponding to the different *Legionella* species are clearly separate and distinct for each taxon, with every identified sequence falling on the same branch of the correspondent ATCC/IMVS reference *mip* gene. Therefore, our dataset can be separated into three major clades, in concordance with previous studies (Ratcliff et al., 1998; Burstein et al., 2016): One clade that includes *L. quinlivanii*, *L. jordanis*, *L. feeleii*, *L. nautarum*, *L. rubrilucens*, and *L. taurinensis*; one clade represented by *L. anisa*, *L. steelei*, *L. pneumophila* (positive control), and *L. quateirensis*; and one that includes *L. londiniensis* and the so-called *L. species* H. Interestingly, the tree shows how *L. rubrilucens* and *L. taurinensis* form a monophyletic group, revealing a high genetic similarity (96.3%).

Concerning the intra-species analysis, the tree reveals the presence of a subclade inside the *L. anisa* principal clade, formed by two isolates sequences (i.e., *mip*-17 and *mip*-49), which share only 96.7% pairwise identity with the *L. anisa* reference *mip* sequence (i.e., ATCC 35392). The *mip* gene of the *L. quinlivanii* isolate shares only 96.2% sequence similarity with its correspondent reference strain (i.e., ATCC 43830).

## Discussion

The role of *Legionella* in human respiratory infections emphasizes the importance of its surveillance in conventional and unconventional artificial environments that could represent a reservoir of infections (Szewzyk et al., 2000).

One of the main concerns during environmental and clinical *Legionella* surveillance is the need of a rapid and sensitive technique that could improve therapeutic and epidemiological choices.

First, it could allow the timely adoption of an appropriate antibiotic treatment, as well as the rapid identification of the source of infection by comparison between strains isolated from environmental and clinical samples, thus enabling the adoption of the correct prevention measures to control the infection. The long incubation time, the poor sensitivity of serological agglutination methods, and the long and laborious molecular techniques have long been the object of scientific discussion, suggesting the need to enhance *Legionella* identification with faster, cheaper, and more sensitive methods. MALDI–TOF MS is nowadays the universal method for microbial identification at the species level in routine microbial identification laboratories. In this study, a comparison between three different techniques (i.e., the agglutination test, *mip*-gene sequencing, and MALDI–TOF MS) was performed in order to assess whether MALDI–TOF MS technology could be a useful and valid tool to identify environmental *Legionella* species strains, reducing analytical times and costs.

The strains included in this study were collected from different artificial water reservoirs, chosen considering the presence of a routine surveillance program (e.g., hospitals and healthcare facilities), and environments where a water safety plan is not required or is missed (e.g., homes, companies, and recreational communities). Starting from the cultural and diagnostic techniques commonly used on *L. pneumophila*, we evaluated the possibility of carrying out species-level identification using MALDI–TOF MS, including *Legionella* non-*pneumophila* isolates. Although some species are linked to the epidemiology of *Legionella* diseases, a standardized diagnostic approach for environmental and clinical samples has not yet been defined. These species are abundant in the environment, but are clinically less associated with human cases due to a lack of culture, a low growth rate, and the poor sensitivity of diagnostic techniques (Fields et al., 2002; Svarrer et al., 2012; Mercante and Winchell, 2015).

All of these limits with regard to *Legionella* non-*pneumophila* species research lead to a misidentification of strains and to an underestimation of the real risk represented by environments in which they can survive and proliferate.

In our study, we reproduced routine laboratory workflows for *Legionella* surveillance, i.e., sampling, standardized culture, and biochemical–serological testing to elaborate an analytical report. Our culture results showed that most of the isolates grew after more than 10 days of incubation. Despite the reference technical guidelines provided by standard institutions, which suggest an incubation time of a minimum of 10 days, in routine laboratory practice, the time of incubation is typically no longer than 10 days, with a loss of isolate growth after this time (Italian Health Ministry, 2015; ISO 11731:2017 Water quality—Enumeration of *Legionella*, 2017).

The growth of colonies on BCYE cys + is the only discriminating element for routine investigations: Isolates with positive growth are subjected to the latex agglutination test and, when agglutination fails, the results are reported in terms of counts of colonies (CFU/L) followed by a generic identification of “*Legionella* non-*pneumophila* species detection.” The misidentification of isolates leads to an underestimation of circulating species in the environment and, consequently, to inappropriate preventive measures to contain the risk.

In the current research, the agglutination test returned positive results for only 34/202 (16.8%) isolates, failing to detect most of the *Legionella* non-*pneumophila* species isolates, i.e., 158/202 (78.2%). This result is related to limited equipment that does not recognize all of the *Legionella* non-*pneumophila* species; therefore, this assay is not suitable for discrimination within the *Legionella* genus, according to Orsini et al. (2011), and highlights the need to establish, in routine analysis, a further technique able to accurately identify these *Legionella* species.

The MALDI Biotyper system, following the manufacturer’s criteria, overall identified 90/202 (44.5%) *Legionella* isolates at the species level, corresponding to all of the species for which reference spectra are present in the database. A further significant number of isolates (*n* = 40) could have been identified by applying only the additional criteria described by Christner et al. (2010); Schubert et al. (2011), and Martiny et al. (2012).

The failed identification of the remaining 72 isolates is easily explained by the absence of the species in the MALDI Biotyper database (Moliner et al., 2010; Fujinami et al., 2011; Dilger et al., 2016).

The version of manufacturer’s database is closed and can be improved only by the manufacturer and it is covered by intellectual property rights. Therefore, the *Legionella* spectra identification produced in the study, could be represent our first “in-house database” for developing *Legionella* MALDI Biotyper application. These isolates were identified successively only by *mip*-gene sequencing as *L. taurinensis*, L. *londiniensis, L. nautarum, L. species H*, *L. quateirensis*, *L. quinlivanii*, and *L. steleei*.

Interesting results were observed among *L. tauriniensis*, as 39/79 isolates were misidentified (with a low confidence level) as *L. rubrilucens*, likely due to the close relatedness of these two species (Lo Presti et al., 1997; Baladron et al., 2006). In the phylogenetic tree, the two species are part of two distinct clades, although the differences between them only account for 3.7% (24 mismatches), regrouping them in the same monophyletic group. All *L.tauriniensis* isolates showed negative or ambiguous agglutination results and their identification was achieved only via the genotyping approach. The misclassification of *L. taurinensis* with other *Legionella* non-*pneumophila* species (i.e., *L. rubrilucens*, *L. erythra*, and *L. spiritiensis*) occurred already in the first characterization of the species, performed on the *16srRNA* gene, that led to considering them as belonging to the same species (Lo Presti et al., 1997; Baladron et al., 2006). The subsequent characterization by the *mip* gene was able to distinguish between four different species. The MALDI–TOF MS approach, which establishes bacteria identification based on proteomic profiles, is likely unable to detect low differences at the genomic level, and thus returned no results. Different studies in the fields of bacteria identification suggest to combine MALDI Biotyper system analysis with Fourier-transfrom infrared spectroscopy (FTIR), which can be used to analyze carbohydrate and glycoproteins compounds, is able to provide a unique “fingerprint” spectrum for each species of bacteria exceeding the limit previously described (Maity et al., 2013; Feng et al., 2020). The three positive controls, belonging to different serogroups (i.e., 1, 3, and 6), were identified only at the species level without serogroup identification, confirming that the MALDI Biotyper system is unable to discriminate between *L. pneumophila* among serogroups.

A concordance between the three methodologies included in this study was observed only for *L. anisa*. Interestingly, only two out of the 52 *L. anisa* isolates (i.e., *mip-49* and *mip-17*) identified by the MALDI Biotyper system with a low confidence level corresponded to the sub-clade exhibiting a difference of 3.3% (20 mismatches) to the principal clade. We could speculate that these genetic differences can cause differences at the ribosomal protein level that could be underrepresented in the MALDI Biotyper system database, which includes nine *L. anisa* reference spectra. However, the differences found for the two sub-clades of *L. anisa* need further investigation.

The comparison of the *mip*-gene sequencing of one isolate of *L. quinlivanii* showed a similarity of 95.91% to the reference strain ATCC 43830, with only 3.8% of differences linked to 23 mismatches.

Considering the criteria adopted by Ratcliff to develop the *mip*-gene database (Ratcliff et al., 1998), we found some discrepancies that could be improved by further investigation.

Finally, we found a different isolate from all of the characterized species, matched to the *L. species* H described by Ratcliff et al. (1998), close to the *L. londiniensis* clade, with differences of 34.7%. These findings will be improved by the whole-genome sequencing (WGS) approach in order to obtain more information regarding the isolates’ characteristics and their evolutionary adaptations in the environment.

These findings show that the MALDI Biotyper system is a powerful tool for the identification of *Legionella* non-*pneumophila* species other than *L. pneumophila*, as demonstrated by its high sensitivity (96.8%) for the species included in the database and its low specifity (63.9%) that could be improved by amplifying the database with species missing or less represented (e.g., only one spectrum for *L. rubrilucens*). The limit of this study is linked to the need to improve the manufacturer’s database and re-evaluation of the identification score criteria that could support the introduction of MALDI-TOF MS into routine clinical and environmental *Legionella* surveillance. The rievaluation of our “in-house database” with the new one from manufacturer represent the areas for future research.

Moreover, the approach used in this study could develop the knowledge regarding the relationship between strains in water distribution systems, in addition to supporting the rapid identification of the source of infection, the match to clinical strains, and the adoption of corrective actions to limit the spread of bacteria and to control nosocomial and community infection.

## Data Availability Statement

The datasets presented in this study can be found in online repositories. The names of the repository/repositories and accession number (s) can be found below: NCBI GenBank (accession numbers: MW021138 and MW052863–MW053066).

## Author Contributions

SC and MP conceived and designed the experiments and wrote the manuscript. MM and LG performed the samples collection and the experiments. SS performed the phylogenetic analysis. AG and MLS performed mip-gene sequencing. MC, PDM, and FB performed MALDI-TOF MS analysis and data interpretation. MBS and MV supply communities *Legionella* isolates. All authors contributed to the article and approved the submitted version.

## Conflict of Interest

The authors declare that the research was conducted in the absence of any commercial or financial relationships that could be construed as a potential conflict of interest.

## Footnotes

1 http://bioinformatics.phe.org.uk/cgi-bin/Legionella/mip/mip_id.cgi

2 https://www.geneious.com
